# The cardinal rules: Principles of personal protective equipment for high-consequence infectious disease events

**DOI:** 10.1017/ice.2023.264

**Published:** 2024-06

**Authors:** Sara K. Donovan, Jocelyn J. Herstein, Aurora B. Le, Shawn G. Gibbs, Elizabeth L. Beam, Christopher K. Brown, Abigail E. Lowe, John J. Lowe, James V. Lawler

**Affiliations:** 1 Global Center for Health Security, University of Nebraska Medical Center, Omaha, Nebraska; 2 Department of Environmental, Agricultural, and Occupational Health, College of Public Health, University of Nebraska Medical Center, Omaha, Nebraska; 3 Department of Health Behavior, School of Public Health, Texas A&M University, College Station, Texas; 4 Department of Environmental and Occupational Health, School of Public Health, Texas A&M University, College Station, Texas; 5 College of Nursing, University of Nebraska Medical Center, Omaha, Nebraska; 6 Division of Emergency Operations, Center for Preparedness and Response, Centers for Disease Control and Prevention, Atlanta, Georgia; 7 Department of Allied Health Professions Education, Research, and Practice, College of Allied Health Professions, University of Nebraska Medical Center, Omaha, Nebraska; 8 Department of Internal Medicine, College of Medicine, University of Nebraska Medical Center, Omaha, Nebraska

## Abstract

In recognition of an increasing number of high-consequence infectious disease events, a group of subject-matter experts identified core safety principles that can be applied across all donning and doffing protocols for personal protective equipment.

In the past decade, the world has witnessed an unprecedented number of high-consequence infectious disease (HCID) events. With an increase in HCID events comes a demand for increased training for healthcare workers on the donning and doffing of personal protective equipment (PPE). The repercussions of insufficient training, inconsistent use, and improper donning and doffing practices of PPE can be severe and are well documented, including the infection of 2 nurses with Ebola virus disease in Dallas, Texas, in 2014.^1^ As we seek to improve capacity for safe management of patients infected with HCIDs, it is time for the isolation care community to achieve consensus on best practices for donning and doffing PPE. We convened a working group of subject-matter experts (SMEs) with experience with HCID outbreaks to develop cardinal rules for PPE donning and doffing. Although the various PPE brands, types, and models available appropriately renders diverse donning and doffing practices to protect workers from occupational exposure to HCIDs, we sought to identify core principles or cardinal rules that can apply across all specific donning and doffing protocols. These principles serve to anchor training and facilitate comprehension in infection prevention and control practice. The resulting list of cardinal rules, while not all-encompassing, is a starting point for further discussions on key considerations across all donning and doffing sequences for preventing HCID transmission.

## Methods

To identify any extant materials on the principles of donning and doffing PPE, we conducted a review of the literature within PubMed and SCOPUS databases in October 2022. The same key words were used for both databases and were combined using Boolean operators as follows: “principles of donning” OR “donning principles” OR “principles of doffing” OR “doffing principles” OR “rules of donning” OR “donning rules” OR “rules of doffing” OR “doffing rules” OR “fundamentals of donning” OR “donning fundamentals” OR “fundamentals of doffing” OR “doffing fundamentals” OR “best practices for donning” OR “best practices for doffing” OR “donning considerations” OR “doffing considerations.” Manual searches of Google and Google Scholar were conducted to supplement these findings and to identify grey literature. Criteria for inclusion were publication in English and a substantial description of key considerations or principles when donning and doffing PPE for HCID response. Literature was not excluded according to date of publication, setting (ie, clinical, nonclinical, etc), or geographic location.

Literature focused primarily on donning and doffing sequences was reviewed but was not included in the final articles used to develop the cardinal rules because sequences rightfully vary with the types and models of PPE utilized. Search results were eliminated in 3 stages: (1) before screening (duplicate records), (2) during screening of title and abstract, or (3) during full-text screening. If the work did not have an abstract, it was screened according to its title and introductory or background text. Our search was not exhaustive; we sought to identify the most relevant works. The authorship group was comprised of SMEs in industrial hygiene, occupational safety and health with a focus on PPE, virology, epidemiology, infection prevention, infectious diseases, and health behavior with a focus on training and education. This author group possesses vast experience responding to HCID events. They were brought together to generate cardinal rules for donning and doffing PPE for HCIDs, informed by the exploratory literature review.^2^ Discussions were conducted through videoconferencing and email correspondence utilizing a nominal group technique.^3^

## Results

**Figure 1.**
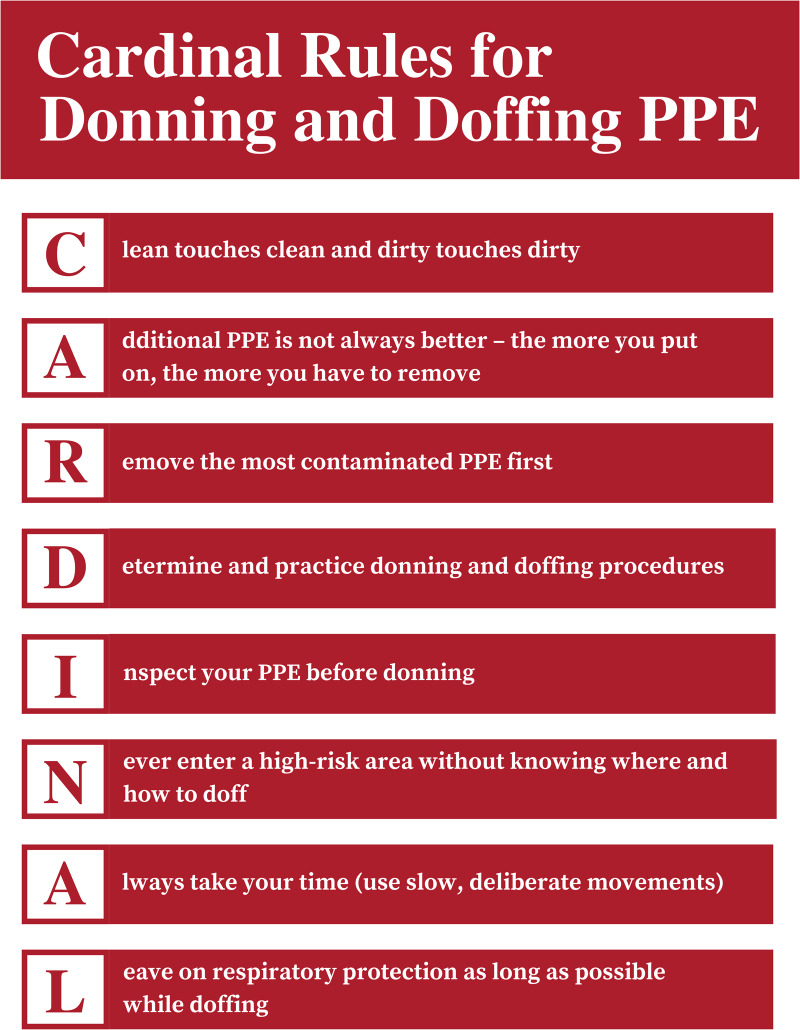
Cardinal rules of donning and doffing: PPE mnemonic.

In total, we identified 117 records by the search of databases, with an additional 6 items of grey literature identified by the supplementary search. Grey literature represented guidance from agencies including the Centers for Disease Control and Prevention and the National Emerging Special Pathogens Training & Education Center. All 123 records underwent title and abstract screening, or screening of the introductory text in the absence of an abstract; 23 articles were reviewed in full. Three works, with publication dates ranging from 2014 to 2020,^4–6^ expanded on the principles of safe donning and doffing and were used by authors as a basis to apply and expand their experiences to produce the cardinal rules (Table 1 and Fig. 1). However, by reviewing the 20 additional full texts, many of these considerations were weaved into the cardinal rules. The rules, in the form of an acronym to facilitate learning retention, are broad and applicable to all levels of PPE, focusing on the fundamentals to promote adherence to established procedures and reduce potential for self-contamination.

**Table 1.** Cardinal Rules of Donning and Doffing PPE

Principle	Explanation
1. Select your equipment based on the risks you will encounter.	More PPE is not always better. The more equipment you put on, the more you have to remove, and the more complex donning and doffing processes become.
2. Never don until you have a well-defined plan for doffing.	There must be established and practiced donning and doffing procedures, with workers able to describe the ‘whys’ behind each step. Confusion or inconsistencies in operations can increase the risk of contamination. Hand hygiene must be performed following doffing, and the doffing area should be decontaminated and must be reset after each doffing event.
3. Prepare; always inspect your PPE before donning.	Inspect all your PPE before donning for tears, defects, or material degradation. Ensure the PPE selected is appropriate for the task and fits (and for respirators, that the person donning has been fit-tested and medically cleared as part of a respiratory protection program).
4. Checklists, partners, and mirrors make for success.	The availability and use of mirrors, checklists, and donning and doffing partners can minimize self-contamination and can ensure that established processes are being adhered to. It is important that the entire body is inspected.
5. Slow is intentional, intentional is safe.	To reduce the risk of PPE breaches or self-contamination, use slow and deliberate movements.
6. Hand hygiene is never wrong.	When in doubt regarding whether you touched something dirty, disinfect your gloves before proceeding.
7. Protect your mucous membranes.	Respiratory protection should be left on as long as possible while doffing to provide protection to the mucous membranes. When doffing, avoid touching the front of the mask or respirator.
8. Clean touches clean; dirty touches dirty.	Do not use contaminated or potentially contaminated gloves to touch clean PPE. Once you have touched a dirty surface of PPE, your gloves are dirty and should only touch other potentially contaminated PPE surfaces.
9. Remove the most contaminated PPE first.	The removal (and perhaps replacement) of the most contaminated items first will prevent the spread of contamination to other items of PPE during the doffing process.
10. When doffing, move from dirty to clean.	Move to cleaner areas as you remove the most contaminated items. There should be clear separation between contaminated, potentially contaminated, and clean areas.

## Discussion

The review of literature indicated an absence of broad, widely applicable foundational rules for donning and doffing PPE, despite an increasing number of HCID events globally. PPE is a critical component of worker safety for HCID care, providing a layer of protection that cannot be overrstated. The application of the cardinal rules, regardless of sequences or types of PPE, in clinical and nonclinical settings, can minimize self-contamination events during infectious disease response, providing greater protection to workers and, ultimately, communities. Although not the focus of this article, it is critical to recognize that core principles accompany PPE donning and doffing sequences (eg, when hand hygiene should occur, disposal of soiled PPE) and that some PPE use was not included in our search that informed the development of these cardinal rules. Moreover, we recognize that the vast number of considerations and nuances for safety around PPE donning and doffing are far too comprehensive to distill into a single set of 8–10 principles. An opportunity remains for the infection prevention and control and HCID communities to continue building on and refining these cardinal rules to maximize this resource for practical use in any setting.
